# Royal power in the market‐oriented society: The Swedish King's consecration of business and corporate elites

**DOI:** 10.1111/1468-4446.13173

**Published:** 2024-11-30

**Authors:** Mikael Holmqvist

**Affiliations:** ^1^ Stockholm University Stockholm Sweden

**Keywords:** consecration, elites, neoliberalism, power, royals

## Abstract

In this paper, I examine how the King of Sweden, Carl XVI Gustaf, systematically consecrates the nation's business and corporate elites who have come to dominate Swedish society during the last decades concomitant with a fundamental transformation from traditional social‐democracy to neoliberalism, that is, a society characterized by the logic of corporations and markets. By promoting the business and corporate elites, the King contributes to strengthening their status and legitimacy in relation to other groups, while at the same time he reproduces his own elite status and image as a “corporate king.” In order to examine this dual elite legitimation, I have studied three major official duties in the King's official role as Sweden's head of state: (a) the awarding of the most prestigious royal medals to corporate leaders; (b) the invitation of these elites to official royal dinners; and (c) state visits, whereby the corporate elites are given a peculiar status in relation to other elite groups. Based on this unique data on the activities of a living monarch, I refute the common assumption among sociologists today that royals, and particularly monarchs, are powerless figures and therefore irrelevant as study objects. By consecrating business and its leaders, monarchs contribute to legitimizing neoliberalism, thus strengthening its hegemony, as well as their own standing. Hence, they are not only symbolic figures, but exercise real power as well.

## 1 INTRODUCTION

Typically among sociologists, royals are thought to be powerless, upholding merely symbolic status in contemporary society, particularly in the Western constitutional monarchies of the United Kingdom, Spain, Belgium, the Netherlands, Scandinavia, Japan, Australia, New Zealand and Canada. Certainly, a few studies have recently been concerned with contemporary monarchies, paying particular attention to these institutions' capitalist and market‐oriented image‐making and character (see Billig, 1992; Clancy, 2021; Prochaska, 1995; Unchanam, 2020); there are also a number of historical analyses of royalty by sociologists, which focus on royal traditions and ceremonies, and the functions of royal courts (see e.g., Bendix, 1978; Birnbaum, 1955; Elias, 2006; Olechnowicz, 2007; Shils & Young, 1953). Among the few sociological studies that exist on royals, even fewer have examined what individual monarchs do on a daily basis in their official roles as their nations' heads of states, and the sociological implications of these activities (cf., Bergman, 1992; Wenander, 2020). Largely, the individuals central to today's monarchies—the monarchs themselves—have been excluded from the narrative, as if they were of no importance and irrelevant as sociological study objects.

However, as suggested by the empirical observations offered in this paper, this appears a spurious position. Instead, royals, and particularly monarchs, can be seen as potentially influential elites in contemporary society, that is, groups with unproportionate access to economic, cultural, organizational and social resources and capital (see Bourdieu, 1996; Cousin et al., 2018; Friedman & Reeves, 2020). Certainly, with the exception of the European micro‐monarchies of Monaco, Luxembourg, the Vatican State and Lichtenstein, and the Middle‐Eastern and Asian kingdoms of, for example, Saudi Arabia, Jordan and Thailand (see Hazell & Morris, 2020; Unchanam, 2020; Yom & Gause, 2012), monarchs such as the British, Dutch, Norwegian and Spanish kings may not (any longer) have access to formal “command positions” as in Mill's (2000) definition of elite status through which they may exert some real impact on people's daily lives. But like many other powerful actors that don't uphold any formal positions, they do have extraordinary access to various economic, social, cultural, organizational and communicative resources, which makes them potentially influential (cf., Cousin et al., 2018; Gulbrandsen, 2019; Holmqvist, 2017; Khan, 2012). For instance, the British monarch is extremely wealthy, owning land and other property, which makes him powerful in an economic and financial sense; and the recently abdicated Queen of Denmark, Margrethe II, was considered a skilled artist who exerted influence on the public debate in Denmark on arts and culture (see Clancy, 2021; Mosegaard Amdisen, 2019).

Most importantly, through their unique socially and morally elevated status and aura of mystery and enchantment (see Billig, 1992; Nairn, 2011), which is linked to a historical idea of royal divinity, monarchs have the potential to consecrate their environments, that is, sanctifying things and people, making them appear “better” and “more worthy” according to certain interests and ideologies, even holy, whereby they both physically and literally can elevate the standing and status of people (see Durkheim, 1973, p. 175; Weber, 1946, p. 262; see also Accominotti, 2021; Khan, 2011; Pargament & Mahoney, 2009). Indeed, the notion of consecration is a central part of the coronation of the British monarch, whereby he or she is anointed with “holy” oil, thus symbolically making him/her an agent of consecration.

One of the key intentional or unintentional activities of contemporary royals is the promotion and legitimation of certain ideas and interests that are associated with society's dominating elites, be it by their presence at various politically and socially prestigious events, such as the opening of national parliaments, their awarding of prestigious medals, prizes and other honors, their speeches, for example, on the occasion of Christmas or New Year's Eve, or their official dinners. As I will try to show in this paper, the common practices among contemporary monarchs, in their roles as heads of states, of (a) awarding prestigious royal medals and orders to “deserving citizens”; (b) inviting people to lavish official royal dinners; and (c) their execution of state visits (see Hazell & Morris, 2020; Unchanam, 2020), are not neutral and apolitical, and are therefore not insignificant from a social or political point‐of‐view. Instead, they can be seen as critical expressions of monarchs' consecrating powers, through which they elevate the social standing of certain people and groups. Hence, consecration is something more than legitimation (see Csaszni Halvorsen, 2024). It's therefore of interest to examine what groups in society are consecrated by monarchs, that is, who are “made holy” in a symbolic sense and thus considered socially and morally superior?

As I will argue, monarchs' unique sanctification of things and people can be regarded as an important source of elite legitimation through which they not only justify their own positions and authority, which is critical to their standing and continued power, but also contribute to legitimizing certain groups and people (see Altermark et al., 2023; Kumar Wariko & Fuhr, 2013), particularly taking into account that their activities are typically the subject of intense media interest (see e.g., Clancy, 2023; Jönsson & Lundell, 2009; Omes & Maclaran, 2015): As in no way before in history, what monarchs do and say are spread to large groups of people, almost instantly; in relation to other elite groups, such as the business elites, academics, cultural elites and political elites, royals are in addition the subject of extraordinary interest by the general public.

More specifically, I examine how the King of Sweden, Carl XVI Gustaf, socially and morally elevates Sweden's business and corporate elites who have come to dominate Swedish society during the last decades against the background of a fundamental transformation of Swedish society from traditional social‐democracy to neoliberalism (see Aaberge et al., 2018; Offer & Söderberg, 2016). By doing so, the King contributes to strengthening these elites' status and legitimacy in relation to other elites, while at the same time he contributes to reproducing his own status and legitimacy as a “modern,” “corporate monarch.” Overall, the King's consecration of business elites can be understood against a background of the global phenomenon of neoliberalism; thus, the behavior of the Swedish King should not be seen as surprising, given the fact that neoliberalism requires elites to promote certain values and ideals that are associated with this ideology, in order to remain legitimate (see Cousin & Chauvin, 2014; Friedman & Laurison, 2019). At the same time, they contribute to reproducing it, thus furthering its overall legitimization (see Altermark et al., 2023; Persson, 2023; Van Zanten, 2018).

## 2 CORPORATE ELITES AND NEOLIBERALISM

During the late 1960s, Swedish social democracy reached its ideological peak, where Sweden's “third way economy,” between socialism/communism and market economy/capitalism was the dominating ideological system. Certainly, Swedish business was the backbone of the economy, but was closely monitored through various welfare‐state regulations. Likewise, the corporate and business elites were heavily taxed in order to accomplish social and economic equally and integration between classes. Overall, their social status was precarious, and they kept their heads down. But all this started to change in the early 1970s; for the first time in many decades a coalition between liberals and conservatives won the government elections in 1976, resulting from public dismay with the social democratic government's economic policies. Inspired by the governments of Thatcher and Reagan in the 1980s, later Swedish governments, both conservative and social democratic, started to gradually turn Sweden into a full‐blown market economy through various deregulations and privatizations (see Aaberge et al., 2018; Benner & Holmqvist, 2023; Offer & Söderberg, 2016), for instance resulting in the creation in the 1990's of one of the world's most deregulated school systems (see Åström Rudberg, 2023).

Indeed, the global shift from welfare state/social‐democratic government toward neoliberal government in the late 1970s and early 1980s altered the criteria of gaining legitimate powers in ways that not only amplified but also re‐arranged social hierarchies, favoring the corporate and business elites at the expense of others (see e.g., Cousin & Chauvin, 2014; Ho, 2009; Kantola & Kuusela, 2019), hence suggesting that elites, depending on how well they are aligned with the dominant criteria of gaining legitimate powers (see Weber, 1978), are not necessarily stable over time (see Mills, 2000; Pareto, 1991).

To be more specific, the market‐turn of societies involved both a structural and an ideological dimension that promoted the standing of business and corporate elites, effectively turning them into important “status groups” in contemporary society (cf., Weber, 1946): Structurally, it implicated financial deregulations, lowered interest rates and inexpensive credit, reduction of capital and corporate taxes and the opening up of the publicly owned welfare sector to private investors and market forces, thus nurturing the creation of new wealth elites (see Beaverstock, 2010; Hall, 2009; Kantola & Kuusela, 2024; Kuusela, 2020). Private enterprises, which earlier were locked into national economies, became able to place their head‐offices where credit was stable and inexpensive and taxes on capital and profits were low (see Harrington, 2016; Luyendijk, 2016). Inversely, states were forced to compete with each other by offering capital investors the best possible business climate: low taxes on capital and corporate profits, low‐cost credit and a combination of high‐quality human capital or low‐priced labor (Jessop, 1992; Offer, 2017; Piketty, 2020).

With inexpensive credit and low taxes on capital and profits, those who already owned capital became increasingly rich, even “super‐rich” (Beaverstock et al., 2013; Hay, 2016), overall favoring the social and political standing of the business and corporate elites (see Atkinson, 2021; Harrington, 2016). Also, new business elite were established, further strengthening this group's ideological dominance: The entrepreneurs, primarily in the finance and tech industries, but also in the new privatized social welfare sectors, particularly in the transforming European welfare states such as Sweden (see e.g., Audretsch, 2007; Brockmann et al., 2021; Christopoulos & Vogl, 2015; Kantola, 2020; Saebi et al., 2019).

Overall, the growing social and economic importance and status of the corporate and business elites challenged established hierarchies based on privilege, replacing them with new hierarchies of “deserving” entrepreneurial competitors (cf., Friedman et al., 2024; Korsnes et al., 2018; Savage & Waitkus, 2021). The spread of new meritocratic ideals (see Mijs & Savage, 2020; Noble & Roberts, 2019; Sandel, 2020), have contributed to that the growing inequalities that have developed in the aftermath of neoliberalization have generally not been perceived as the rebirth of the class societies that melted away in the post‐war societies (Harvey, 2005; Piketty, 2020); they have instead largely been seen as fair or even good: People should get what their merits imply that they deserve has become the new mantra (Friedman et al., 2024; Kennedy & Power, 2008; Khan & Jerolmack, 2013).

Overall, then, neoliberalization is closely aligned with the social, political and economic rise of the corporate and business elites during the last decades, as new “status groups” (cf., Mills, 2000; Weber, 1946). Previously, they had ranked low on the social ladder (see Alfani, 2023; Khurana, 2007; Röbken, 2004), but through concrete neoliberal policies, such as privatizations and deregulations that have favored the accumulation of wealth to a few, they have gradually become the new masters of the universe, socially, morally, aesthetically and economically (see Alvesson, 2011; Holmqvist, 2023).

## 3 METHODS

In order to examine the consecrating activities of the Swedish King, which forms part of my larger study of the King as Sweden's head of state (see Holmqvist, 2023), I have studied three major official duties in his role as head of state: (a) the awarding of royal medals as national honors to “meritorious citizens,” that is, persons that are considered by the King as having done substantial contributions to Sweden as a nation, be it in arts, music, literature, academia, business, or some other field; (b) the invitation of “meritorious citizens” to official royal dinners; and (c) state visits, where the King officially represents Sweden, abroad and at home.

Of course, during a typical year the King does many other things in his role as head of state, for instance meeting the prime minister and other government officials for “information purposes,” as well as meeting with corporate, religious and political leaders; he visits government authorities, sports activities, cultural events, businesses, clubs and associations, universities and other institutions of higher education, military entities, award prizes and scholarships, and he inaugurates buildings and bridges, etcetera. But, the awarding of royal medals, the hosting of royal dinners and the performance of state visits are particularly relevant to concentrate on as they are recurrent and symbolically important for his official function as head of state in Sweden as a constitutional monarchy (see Bergman, 1992). Moreover, these activities are standard practices by most heads of states, be they monarchs or presidents; thus, they are not unique to Sweden but offer an empirical ground for comparisons between nations.

Most importantly for my choice of these three activities, are their obvious consecrating potential: Awarding someone a prestigious royal medal is essentially about officially promoting this individual, socially and morally, almost giving him or her a divine status that exceeds any human accomplishments through the extraordinary pomp and circumstances associated with royal medal ceremonies (cf., Altermark & Johansson, 2024); royal dinners and state visits similarly illustrate the consecrating power of monarchs in creating “sanctified actors” (cf., Nairn, 2011; Shils & Young, 1953). For instance, during official dinners and state visits, the King is dressed in royal military uniform, wears orders, medals and grand stars, and is surrounded by court people who are dressed in old‐fashioned and royal clothes, all in all giving the setting a unique, majestic character. In addition, as the ceremonies are usually closely monitored by the media, they become powerful instruments for the King's promotion of certain groups and classes in society at large (see Clancy, 2023; Jönsson & Lundell, 2009; Omes & Maclaran, 2015).

Overall, by being offered a royal medal, or by being invited to a royal dinner, the King aims to legitimize and promote the standing of that person, and what he or she represents, while at the same time acting to legitimize his own position and status. Thus, by examining who gets royal medals and who are invited to royal dinners, we can get an understanding of what kind of values and ideas a monarch aims to idealize and promote, in his or her role as head of state. Similarly, by examining the programs of state visits, we can get an understanding of what kind of ideologies are promoted by nations today, seen through the activities of their respective heads of states.

The data for the awarding of royal medals and the royal dinners were retrieved from the Swedish Royal Court's website, where this information was available for the years 2010–2020. For earlier years, the data was available in the annual reports from the court, which were accessible at the Swedish National Library. My categorization of people who appeared in the official documents were also based on public websites. For the state visits examined, the data was available through the archives of the Swedish Foreign Ministry.

To be more specific, for the awarding of the 426 royal medals between 1980 and 2021, I first summarized them for each year in a word document. The next step was to categorize the recipient and his/her profession, which was based on the information given in the document “Hovkalendern,” issued by the Royal Court. Most people that I identified as “business and corporate elites” were presented with such titles as “Corporate Executive Officer,” “Bank Executive,” “Managing Director,” “Investor,” and the like. Some people had been given the title “Director” (in Sweden, usually associated with business activities), but were professionally in another sector. This was, for instance, the case of a national museum director, which I therefore did not categorize as business or corporate elite. After having categorized all recipients, based on their professional and/or academic titles, I then simply counted the number of people in each category, and finally compared the numbers with one another. For the historic comparison with the two previous monarchs, I collected information on their awarding of royal orders through a periodical called “Statskalendern,” for a select number of years that would reflect a longer period of time. Based on the information in that document on people's professional and/or academic titles, I proceeded in a similar way.

For the royal dinners, I downloaded guest lists from the royal court's website; when they were not available, an official at the court provided me with the requested information, for example, the guest list of the royal wedding luncheon in 1976. For each dinner, I categorized people according to the professional and/or academic titles that was presented in the documents. In some cases the titles appeared incorrect (as in the case mentioned above on the royal medals), which prompted me to make further inquiries on that individual in order to correctly categorize him/her. For some of the guest lists, invitees were only presented with “Mr.,” “Mrs.,” or “Miss”; in that case I did investigations on the web in order to get information on their professional and/or academic background and position. This was particularly common for the personal friends of the King that had been invited to his official birthday dinners.^1^ Based on the categories made, I summarized the number of individuals and could compare each sub‐category with one another.

Finally, for the state visits, which were different in terms of data in relation to the medals and the dinners, I retrieved the full programs for 10 state visits, which I had chosen based on their variation in terms of economic and social character. Examining the programs, I could conclude qualitatively that they were very much focused on serving the interests of business and their elites. This was, for instance, marked by the intense presence of large business delegations consisting of national corporate elites during each visit; but also information I gathered from the websites of the royal court and the Swedish government, where they explained the aims and ambitions with each visit. I also collected data from a website called “Mediearkivet,” through which I could download newspaper articles and other media data on each state visit, which further confirmed the business‐oriented character of them.

The data for the three categories—medals, dinner and state visits—differ to some extent, which has to do with access to material in archives and websites. My purpose with this paper is to analyze how Sweden's king, in his role as the nation's head of state, expresses neoliberalism through three of his most important activities, which can be understood against a background of Sweden's gradual market‐turn during the last decades. Rather than relying on one year only, I have included several years, in order to be able to better substantiate that claim, but there has been no ambition to track the Swedish monarchy's neoliberalization during the King's more than 50 years of reign, which would have required a partly different empirical approach.

## 4 FINDINGS

Before reporting on the King's three consecrating practices, I will first offer a short historic background on him and his role as Sweden's head of state, which aim to contextualize the ensuing observations.

King Carl XVI Gustaf ascended to the Swedish throne on September 15, 1973. At the time he was the world's youngest king, 28 years old. He has testified in several media interviews how insecure he felt about his new role: It was a time of great turbulence and stress for the Swedish monarchy, where strong voices were heard in society at large, and among leading social‐democratic and communist politicians, to turn the 1000‐year monarchy into a republic. Overall, the institution, which was dominated by aristocrats and their ethos of privilege based on class and inheritance (see Bendix, 1978; Weber, 1946), was regarded as a symbol of inequality and illegitimate powers by many (see Bergman, 1992; Wenander, 2020). A new constitution was about to be decided by the Riksdag (Swedish Parliament), where all the formal powers of the monarch was to be taken away from him, reducing him to a mere symbol of the nation, not even allowed to formally appoint prime ministers or sign laws any longer (which still today most other monarchs do). In the 1973 proposition from the social‐democratic government for a new constitution, the following was stated by The Minister of Justice: “The Head of State should not be granted any authority whatsoever, which means that he can exercise power (…) The Head of State's tasks are of a representative and ceremonial nature.”

The new constitution specified very few formal duties for the new monarch, limiting them to a handful of meetings yearly with the prime minister and the government for “information purposes,” welcoming new ambassadors to Sweden and saying goodbye to departing ones, opening the Riksdag in September every year, and a few other tasks. Early on, the King and the royal court therefore concluded that in the absence of a detailed and extensive “job description” that could guide his activities, the King had to “invent himself” (see Eliason, 2010). In collaboration with two of his friends and advisers (Fredrik Palmstierna and Tom Wachtmeister), both senior corporate CEO's, he decided his formal motto would be, “For Sweden—With the Times,” which has been an important guiding star for his activities and statements ever since. The motto's intention, according to the King himself as expressed in several newspaper interviews, is that he should act in accordance with the dominant ideas and beliefs of Swedish society, that is, he should be a flexible and adaptive head of state in terms of what he does and says, and not a conservative or reactionary King.

In formulating the new royal motto, and in outlining the first constituent years for the young King, Wachtmeister was appointed First Court Marshal, with formal control of the King's program, and thus with great power of how the King would be presented to the public. Other people with business experience were also appointed by the King to influential positions. “Exit the aristocrats and militaries, enter the entrepreneurs and the executives,” appeared to be the dominating policy. Overseeing it all was the Wallenberg family, Sweden's foremost financial dynasty (see Wetterberg, 2014). The family upheld long historic ties to the Royal Family, dating back to the late 1880s, when their bank was saved from bankruptcy by King Oscar IIs personal economic help (see Bäckström, 2014).

Turning to the business world and its leaders for guidance in how to reinvent the monarchy (see Hobsbwam, 2012), probably appeared natural for the King and his court: the business and corporate elites had remained strong supporters of the monarchy during the “difficult years” during social democratic rule, but they also appeared to be the most legitimate and relevant group to associate with given the gradual market‐turn of Swedish society. In addition, the King's closest advisor in the royal family, his uncle Prince Bertil, who acted as the King's mentor in the absence of his deceased father, was already very active in promoting Swedish industry abroad, and had participated in numerous business trips, paving the way for the young King on how to act and think as a “modern” monarch (see Lindqvist & Tarras‐Wahlberg, 2006). And as a Crown Prince, part of his education program, as organized by the royal court, was to do internships at Swedish corporations, which also probably played a role in the early socialization of him as a future “corporate king” (see Ramel, 1994).

Hence, surrounded by a group of senior business and corporate executives, and given the increasing neoliberalization of Swedish society during the 1970s and 1980s, a certain environment of beliefs and values was created for the young and inexperienced King. In order to remain legitimate, associating with business and its elites probably appeared a “natural” survival strategy (cf., Billig, 1992; Clancy, 2021; Unchanam, 2020), but was also in tune “with the times.”

Breaking with royal tradition to marry within princely houses, the King even married a woman from a wealthy business family, Silvia Sommerlath from Germany, who then became queen; also his closest sister, Princess Christina, married a business man. This matrimonial strategy has even remained for the next generation: The King's first‐born child and the future Swedish monarch, Crown Princess Victoria has married a Swedish business entrepreneur, and his other daughter, Princess Madeleine, an American investment banker. His son, Prince Carl Philip has gone into business himself, as have, by the way, even the King, by owning and managing a large farm focusing on breeding and selling high‐quality livestock. The business and entrepreneurial character of the Swedish royal family can also be illustrated by their members being active players on Swedish and international stock markets, investing their fortunes in various industries and companies (see Lyrevik, 2018).^2^

Haunted by the specter of anti‐royal sentiments during the early 1970s and the clear risk of the abolishment of the monarchy by the dominant Swedish social democratic party, the King and the court appeared desperate to appear modern and relevant, “with the times” as the King's motto said—and they obviously found an ally in the business and corporate world. But, the benefits of this liaison were mutual: As we shall see, the King has “paid back” to the business elites by systematically consecrating them during his 50 years' of reign, thus promoting their standing and power in Swedish society.

### 4.1 Consecrating business elites through the awarding of royal medals

In his role as Sweden's head of state, the King awards royal medals twice a year. The awarding of royal medals is not anything formally required by the Swedish state, it is something the King does by his own initiative. Of all official medals awarded in Sweden, perhaps excluding the Nobel Prize, the King's medals are seen as the most prestigious and desired. They are thus vital instruments for the King to promote and consecrate certain spheres in society, and its representatives. Recipients of royal medals are formally decided by the King upon suggestion by a committee at the royal court.^3^

**FIGURE 1:**
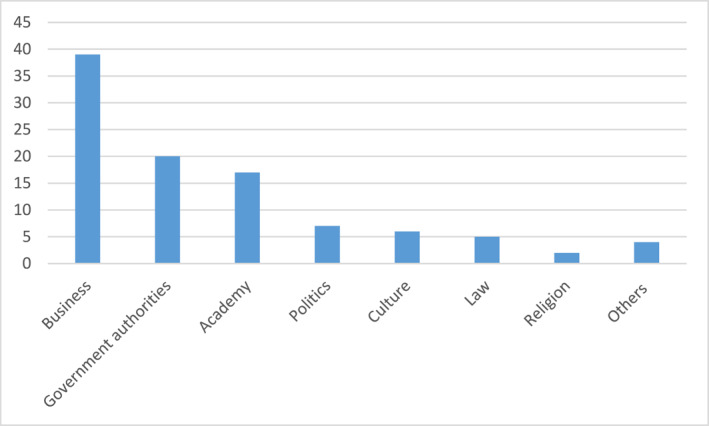
Distribution of royal medals to various elite groups (in percentage).

In examining the awarding of the King's medals during a period from 1980 through 2020, which aim to offer systematic data on his awarding of medals during several years, I have concentrated on the two most prestigious medals, in total the awarding of 426 medals.^4^ With few exceptions, these two medals are given to leaders and elites in various sectors in society, such as business leaders, university presidents, director generals, senior politicians, directors of national arts museums, concert halls, and operas. The medals are distributed according to the following (see Figure 1).^5^

For the dominating category “Business” with its 39% share of medals, the recipients are primarily senior decision‐makers and executives in the corporate world, but also leading entrepreneurs and capital owners. In the documents I retrieved from the royal court, many of them have titles such as “CEO,” “Chairman of the Board,” “Business Person,” “Investor” and similar titles from business and finance. Several are well‐known Swedish corporate leaders. Traditional heavy Swedish industry, for example, automotive, forestry and mining, dominates, as well as bank and finance—the latter well illustrated by the awarding of medals to several members of the Wallenberg financial dynasty. New industries are also represented, such as IT/technology and gaming, however, at a smaller scale. Of the persons that were included in this category, only 13.5% were women. Hence, male corporate and business elites is the largest single group of recipients of the two most prestigious royal medals, thus stressing a very biased consecration in terms of gender.

It should be noted that for all groups except for Business, there is a formal logic in the awarding of the royal medals, as these are normally given to present or previous senior representatives of the Swedish state, be they government directors of authorities (the category “Government authorities”), or presidents and directors of national and state‐owned universities, theaters and museums (the categories “Academy” and “Culture”). These medals are typically given ex officio. The fact that this is not the case for the business and corporate elites, further stresses the King's ambitions to primarily promote corporate and business leaders.

In order to make some historic comparisons, I have also collected data on who were bearer of the most prestigious royal orders during the reigns of the King's two predecessors, Gustaf V (king 1907–1950) and Gustaf VI Adolf (king 1950–1973). This refers to the years 1919, 1929, 1939, 1949, 1959, and 1969, which I have chosen for illustrative purposes.^6^ For the year of 1919, only three senior business persons (two of them being members of the Wallenberg family) were amongst the holders of a prestigious royal order (amongst a total 141), that is, 2%. For the year of 1969, however, the number of business people had risen to 15 amongst a total of 180, that is, 8%.

Hence, although there was an increase in the number of business and corporate elites as holders of the most prestigious royal orders from 1919 to 1969, the situation was still dramatically different than compared to today: During the last decades, which coincides with Sweden's market‐turn, there has obviously been an increasing intensification of royal consecration of business and its elites. By awarding relatively many prestigious royals medals to corporate and business elites, they are given a particular social standing by the head of state, contributing to consecrating their ideals and perspectives, namely neoliberalism.

Legitimizing and promoting values and actors associated with this ideology is, however, fully reasonable given the King's official motto, to act “For Sweden—With the Times,” which suggests the King should constantly adapt his behaviors to the dominating beliefs in contemporary Swedish and global society, which today is neoliberalism: As already suggested, the King's more than 50 years of reign, and following the global market‐turn of economies during the last decades, Sweden has fundamentally transformed from a traditional social‐democratic country, to a country where markets regulate important sectors, such as school and education, the elderly care, and health care, and where the corporate, financial and business elites have come to play an increasingly significant role economically, socially and morally. Hence, the Swedish King, contrary to what is perhaps expected from a monarch who is usually associated with tradition and the upholding of conservative values, has mainly followed the stream; the awarding of prestigious royal medals to business elites suggests he doesn't oppose the market‐orientation of contemporary Sweden or at least tries to balance it to other ideals and ideologies; on the contrary, he legitimizes and promotes it.

The King and the royal court are not alone in awarding business elites medals. The King also presents such honors in other contexts, for example, at the annual meeting of the business‐dominated The Royal Swedish Engineering Academy; further, the prestigious Royal Patriotic Society's (*Kungliga Patriotiska Sällskapet*) most desired medal is called “The Business Medal” and is presented annually to successful company executives and entrepreneurs by a member of the royal family. In this way, Sweden's royals contribute to the legitimation and consecration of business and corporate elites, in both a social and cultural sense.

As suggested by the above reported data, the distribution of the awarded royal medals well reflects Sweden's neoliberal character today, which is the result of a gradual transformation of its economy during the last decades from social‐democracy to market‐liberalism.

### 4.2 Consecrating business elites: Invitation to royal dinners

Next, I will turn to the King's royal dinners, offered at the extravagant Royal Palace in Stockholm, an ancient building of great historic and symbolic importance, and the largest city‐palace in the world.

I have examined the two main dinners that take place twice a year: These are the “National Dinners” (*Representationsmiddagar* in Swedish), and the “Sweden Dinners” (*Sverigemiddagar* in Swedish). The data was retrieved from the Royal Court's website, where guest lists of all royal dinners are publicly available. Typically, guests at the “National Dinners” are leaders on a high level in Swedish society, that is, the power elites. Guests at the “Sweden Dinners,” where a number of persons from each region in the country are invited, typically include regional and local elites.

In addition, I have examined the official dinners given by the King on the occasions of his 50th and 70th anniversaries in 1996 and 2016, respectively.^7^ These dinners are particularly interesting as they include the personal friends of the King as well, which thus can indicate his personal preferences in terms of participants. As already suggested, a royal dinner is typically ranked very highly in Swedish society; to get invited to the Royal Palace by the King and the Queen is normally considered a great honor and is formally offered “meritorious citizens,” that is, people who are regarded as being of extraordinary importance to the nation, simply put the nation's elites.

**FIGURE 2:**
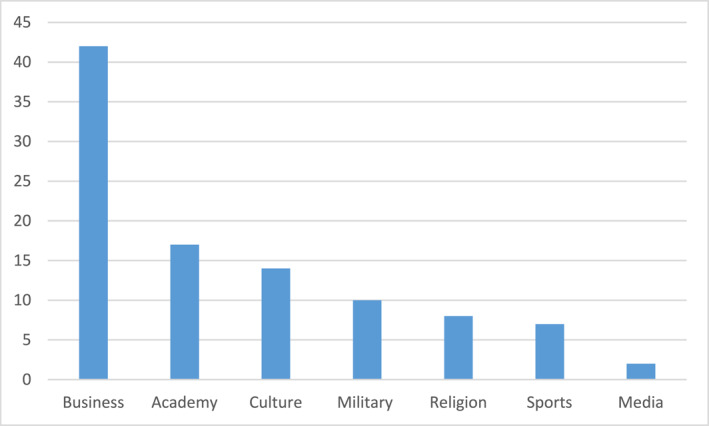
Invitees National Dinners (in percentage).^9^

For the seven “National Dinners” that I have examined (years 2005, 2010, and 2015–2019), in total 405 persons,^8^ the distribution of invitees is the following (see Figure 2).

Thus, there's a clear dominance of corporate and business elites over other groups; by far they consist the largest one, indicating their peculiar status in contemporary Sweden.

**FIGURE 3:**
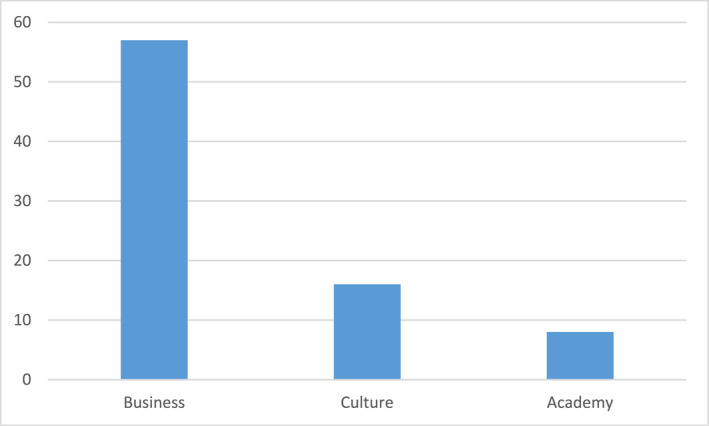
Invitees Sweden Dinners (in percentage, three largest groups).^11^

This picture is even more pronounced when examining six “Sweden Dinners” for the years 2013–2019, a total of 333 persons.^10^ The three main groups of invitees are the following (see Figure 3).

Compared to the “National Dinners,” the “Sweden Dinners,” are even more telling when it comes to the choices and preferences by the King, as there are no formal expectations for this event on whom to invite. As a result, they more strongly signify the way the monarch acts, by inviting relatively more people from the business sector, than from other spheres in society.

It is not known publicly how the selection of invitees to either the National Dinners or the Sweden Dinners take place at the Royal Court; but the distribution well reflects the character of the King's royal motto, “For Sweden—With the Times.” To act “with the times” in a society that has increasingly become all the more market‐oriented suggests that relatively more people from senior positions in the corporate world should be invited than from other spheres in society. This, in turn, will obviously contribute to reproducing the power and status of the business and corporate elites, and the way they legitimate their positions and status.

**FIGURE 4:**
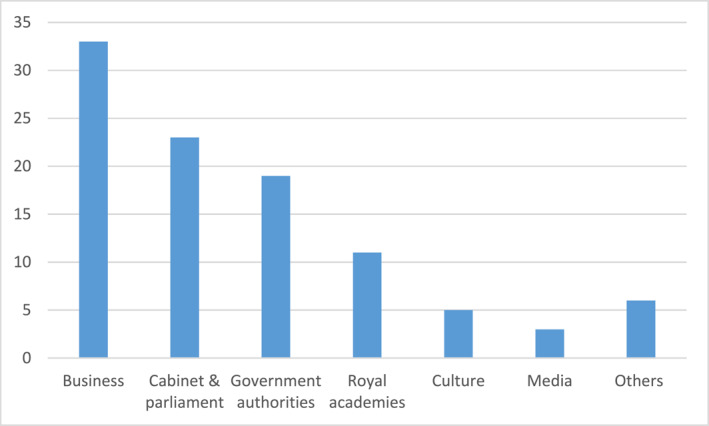
Invitees as “official guests” at the King's 50th anniversary dinner (in percentage).^12^

I will now report on the King's official dinners on the occasions of his 50th and 70th anniversaries. For the first dinner, the guest list discriminates between the following:

(a) *Family and relatives*, which mainly consists of the Royal Family and other relatives in Sweden and abroad (82 couples or individual persons);
(b) *Official guests*, that is, leaders from various spheres in Swedish society—the country's power elite (97 couples or individual persons). This category consists of the following (see Figure 4).

**FIGURE 5:**
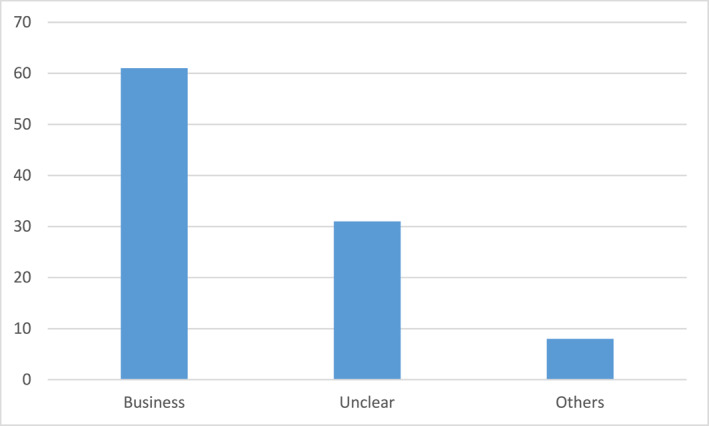
Invitees as “friends” at the King's 50th anniversary dinner (in percentage).^13^

**FIGURE 6:**
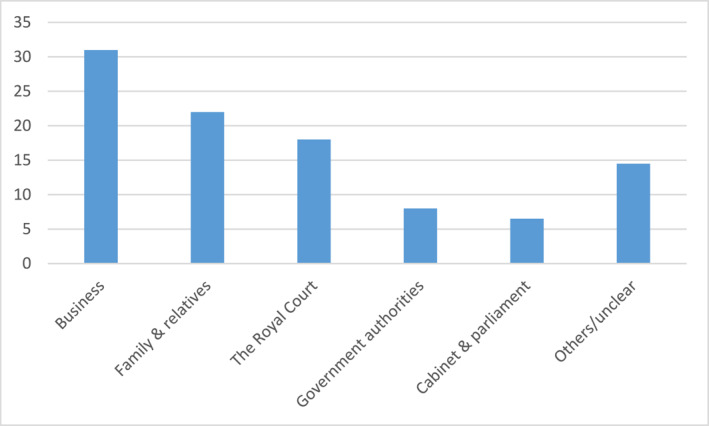
Summary of invitees at the King's 50th anniversary dinner (in percentage).

Hence, among the “official guests,” business elites dominate;

The guest list also consists of officials of the royal court (67 couples or individual persons), which I, however, leave without further analysis. Of more interest, however, is a last category of guests at the King's official 50‐years birthday party, namely his friends, (in total 135 couples or individual persons). For this category, the following distribution is at hand (see Figure 5), well reflecting the dominance of the business elites in being invited to prestigious royal dinners:

Summarizing all categorizes for this event, the following distribution can be reported (see Figure 6).

**FIGURE 7:**
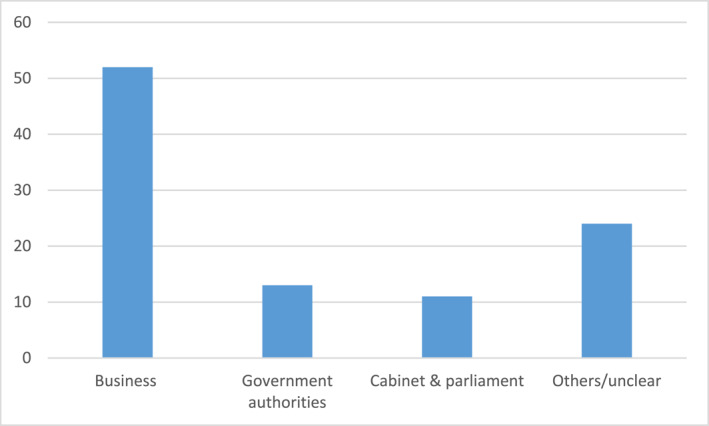
Summary of invitees at the King's 50th anniversary dinner, excluding relatives and the royal court (in percentage).

If excluding family/relatives and court officials from the guest list, who are people on the “inside” of the royal court, the following distribution is given (see Figure 7).

Thus, among those invited to the King's 50th anniversary dinner, there's a clear dominance of the business and corporate elites over other elites; the distribution expresses well the relatively strong consecration by the King of elites in business, contributing to consecrating their status and standing.

**FIGURE 8:**
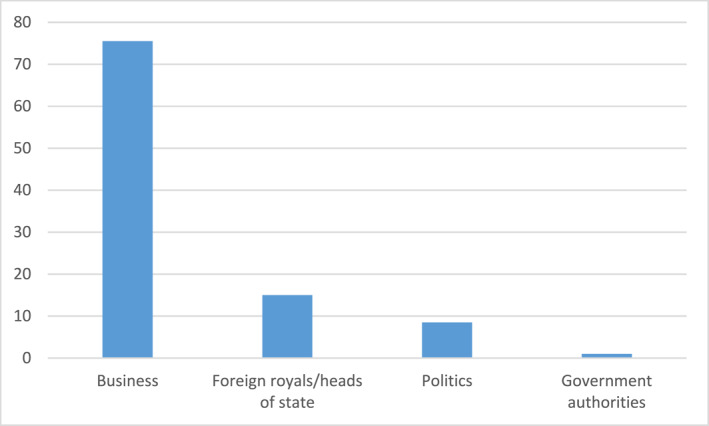
Invitees at the King's 70th anniversary dinner (in percentage).^14^

Without going into details, I can also report that an even stronger presence of business elites was at hand for his official 70th anniversary dinner, 20 years later in 2016. Excluding family members and relatives as well as members of the royal court, the distribution of guests was as follows for both “official persons” and “personal friends” (see Figure 8).

Once again, business elites dominate among the people who are invited to royal dinners at the Royal Palace in Stockholm, and in this way become the target for the King's consecration.^15^ These dinners, including the National Dinners and the Sweden Dinners, are relatively recent occasions, albeit the 50‐years dinner was already in 1996.

As a limited example of a changed character of the guest lists, it can finally be interesting to compare the above reported data with the participants at the royal wedding luncheon in 1976, that is, a prestigious event during the very early phase of Sweden's market‐turn. During this luncheon, celebrating the young King Carl Gustaf and his bride Queen Silvia, only 2 out of 151 persons invited were business people, the smallest of all invited elite groups at that time. Thus, during the King's early phase of his reign, when Sweden was still firmly a social‐democratic country, the business elites had a very modest presence on this occasion. Certainly, my intention is not to analyze the historic transformation of the Swedish monarchy, but this data is still indicative of a larger shift in terms of what groups in society are consecrated by the monarch.

### 4.3 Consecrating business elites through state visits

My next and final data on the way the King contributes to consecrating corporate and business elites in Swedish society, is his participation in official state visits. I have examined the official programs of 10 state visits that represent different parts of the world, sizes, and different economic and social standards, retrieved from the archives of the Swedish Ministry of Foreign Affairs, and the websites of the Government and the Royal Court.^16^

State visits are recurring activities for the King and can be seen as part of his formally regulated duties in accordance with the current constitution. They are very important for understanding the King's role and importance as head of state in today's Sweden and his exercise of power through his royal consecration, not least because they are regularly noticed by the media. Formally, the government is responsible for the choice of countries to visit or to invite, and for the content of the programs. The practical planning is done by the Ministry of Foreign Affairs in close collaboration with the foreign country and with the Royal Court.

There's obviously a large variation in terms of economic, social and cultural factors when considering the countries that I have examined, and there is also a variation in terms of themes, events and invited people for each visit. But, a common observation despite these differences, is that state visits are to a large extent organized to promote business and trade, reflecting the fact that Sweden is a country strongly subject to corporate interests.^17^

Most clearly, this is manifested by the close collaboration between the organizers of the visits, and the organization “Business Sweden,” a joint venture corporation between the Swedish state and national business interests. For all outgoing state visits, a business delegation participates (normally 30–50 high‐ranking corporate executives from Sweden's largest multinational corporations); this is normally not the case for any other elite groups in society such as, for instance academy or culture. As was stated in a press release by Business Sweden on the occasion of the visit to India in 2019:

A business delegation with about 50 Swedish companies and organizations is visiting India 2–4 December, in connection with the Swedish state visit. The business delegation will establish contacts and explore the conditions for deepened business relations with Indian business representatives.

Through the dominance of business both in terms of activities during the state visits, and in terms of people that participate in the delegations, the state visits contribute to making business a symbolically important activity in society: It's primarily not about making business deals, but about legitimizing and consecrating business, and its elites. As the Chairman of the National Business Organization said in a tv‐interview in 2008:

Of course, no deals are done during these types of trips, but you make connections and relationships and often get to meet people you wouldn’t otherwise get to meet. I think everyone who gets the opportunity to travel with the King sees it as a great privilege in that it is something very special.

The King's state visits undoubtedly fulfills an important status‐promoting function for the business elites, where they are offered the opportunity to meet him in both formal and informal situations during several days, and to socialize with other (business and corporate) elites, which is also the reason why senior business representatives repeatedly describe the King's activities within the framework of state visits as important and urgent; by “opening doors” to business and political elites in other countries, the King further contributes to reproducing Swedish business and corporate elites and their ideologies and perspectives. As the King said himself in an interview in 2013:

It’s a lot of fun to be involved and have the opportunity to open doors, and representatives of the business community get the opportunity to meet people they otherwise wouldn’t be able to. It’s great fun. I don’t know if it leads to anything. I would be insanely happy if it leads to business or collaboration in the future, then you feel that you have been involved in enriching and helping Swedish society and the economy, and that’s fun.

**FIGURE 9:**
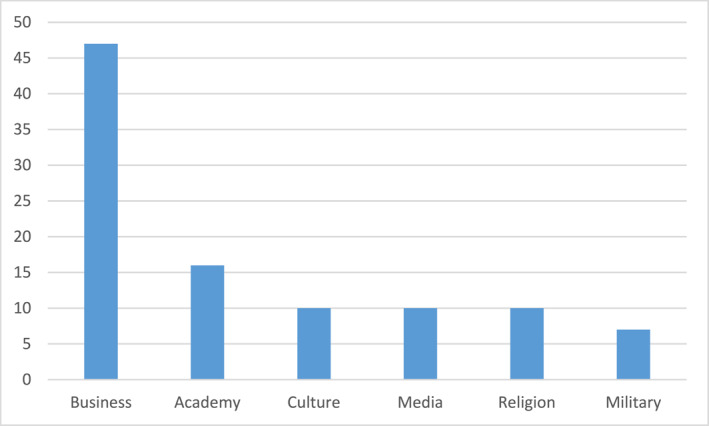
Invitees to eight state visits dinners, excluding state officials (in percentage).

During state visits, there are always so‐called gala dinners, where representatives of various sectors in society are invited. I have examined eight gala dinners that have taken place at the Royal Palace in Stockholm during 2008–2019.^18^ The number of invitees amounted to 360 persons. The largest group of participants was politicians (37%), mainly cabinet members and members of parliament (which are always invited ex officio in great numbers on the occasion of state visits), followed by senior business and corporate people (18%), and senior government authority officials (17%); the latter also typically invited ex officio. Hence, in comparison with elite groups not formally linked to the Swedish state, and thus not invited ex officio, business and corporate elites dominated, the other groups being academic elites (6%), cultural elites (4%), media elites (4%), religious leaders (4%), senior militaries (2.5%), and others, for example, leaders in national social welfare and senior judges, which are less than 2% each, totaling 7.5%. Excluding the political and government authority elites and the category “others” well indicates the privileged role business elites are given in relation to other major elite groups when it comes to participating in gala dinners on the occasion of state visits (see Figure 9).

For my analysis of these and other state visits (see above), I did a detailed analysis of each program. It's not possible to report in full about them here due to limitations of space, but suffice it to say that business activities dominated much of the agendas. The incoming state visit from South Korea in 2019 is a representative case. The visit was described in the following way on the Swedish government's website:

At the invitation of H.M. The King the President of the Republic of Korea Moon Jae‐in visits Sweden together with his wife Kim Jung‐sook on 14–15 June. The president is accompanied by several ministers from the South Korean government as well as a large business delegation.

After the welcome ceremony at the Royal Palace, where the King and Queen together with representatives from the Swedish parliament and cabinet as well as the armed forces greeted the South Korean presidential couple, the president visited the speaker of the parliament, and then had lunch with the royal couple. After lunch, the King and the president headed to Ericsson, Sweden's leading IT‐company, for a visit of its latest facilities. After this visit they jointly participated in the “Sweden‐Korea Business Summit” together with 200 representatives from corporations, startups and investment bankers from Sweden and Korea. At the gala dinner in the evening, a large group of business people participated. Day 2 started with a meeting between the South Korean president and Sweden's prime minister, followed by a visit with the King at Norrsken, a business organization dedicated to innovation and entrepreneurship. According to the document from the Ministry of Foreign Affairs that described the program, Norrsken is an organization that promotes the idea that entrepreneurship and business are the best approaches to address many of the world's most pressing issues and problems. After the visit to this market‐liberal institution, which the King, through his presence contributed to consecrate and legitimize, the King and the president participated in a memorial service for Swedish soldiers who fought in the Korean war. After yet another dinner, with a high level of representatives from the business community, the visit was terminated.

It should be noted that although many Swedish companies regularly participate in state visits, as does the industry organization Swedish Business, many do not; I requested detailed information from the organization Business Sweden about which companies usually participate, broken down by industry, as well as what level of representation occurs (CEO, chairman of the board, etc.), but Business Sweden didn't provide me with such information, for unknown reasons. However, it appears from the content of the state visits I have examined that those companies that participate in state visits are established large companies with commercial interests in the countries in question, not least companies within the so‐called Wallenberg sphere. Business elites, with the Wallenberg family at the forefront, are also usually well represented; however, as a rule, not any entrepreneurs in new economic sectors.

In summary, it can be concluded that the King, in his role as head of state in connection with state visits, has a significant commitment to the private business world, which is marked both by the visits that are carried out, as well as the presence of senior business representatives. During state visits the King is not active in any business‐creating activities; he appears instead as *Hermes*, the merchant's god and protector, which is essential for his image as head of state in a market‐oriented country. Thus, he consecrates the business and corporate elites and their status, which for business is the primary purpose, rather than any commercial results.

## 5 CONCLUSIONS

Previous sociological research on the reproduction and power of business and corporate elites in contemporary neoliberal society have mainly concentrated on their lifestyles in exclusive neighborhood and residential areas (see Atkinson, 2021; Holmqvist, 2017; Wiesel, 2018), as well as socialization patterns in prestigious clubs and unions (see Cousin & Chauvin, 2014; Friedman & Laurison, 2019), and certain educational institutions (see Stevens, 2009; Van Zanten, 2018), particularly the “elite business schools” (see Holmqvist, 2022; Huzzard et al., 2017; Schleef, 2006). But so far, monarchies and individual monarchs have largely been neglected as study objects, as if they were of no importance (cf., Clancy, 2021; Unchanam, 2020).

In this paper I offer data on how the currently longest serving monarch in the world, King Carl XVI Gustaf of Sweden awards the Swedish business and corporate elites prestigious royal medals and invites them to lavish royal dinners relatively more often than other elites, as well as promotes their standing through and during state visits, thus contributing to consecrating the ideology of neoliberalism, that is, a society characterized by deregulations and privatizations of national economies (see e.g., Offer, 2017; Piketty, 2020), and the social, economic and moral dominance of the business and corporate elites (see Harrington, 2016; Hay, 2016; Kantola & Kuusela, 2024), overall manifesting them as critical status groups (see Weber, 1946). Hence, contrary to the established idea of a monarch in a Western constitutional democracy as an apolitical figure, stripped of any real influence and power, the Swedish King contributes to socially and morally elevating, and legitimizing, certain groups, and their ideologies.

Aligning with royals, particularly monarchs, can thus be a very good idea if one wants to promote one's own perspectives and interests, as is the case of the Swedish business elites. Not only can a monarch fulfill the role of legitimizing their standing and status, making their ideology appear honorable and good, much in the same way as elite business schools and similar economic institutions contribute to doing (see Holmqvist, 2022; Schleef, 2006); a monarch can also be used as an instrument of legitimization of how society should be managed and organized from a moral, social and economic point‐of‐view, in this case according to neoliberal principles and values. To this extent modern European constitutional monarchies as the Swedish one, which are not formally or traditionally considered economic institutions (such as markets, corporations and businesses), can be understood to be, by following Weber's (1949) typology of economic institutions and their relation to “non‐economic” phenomena, as both “economically conditioned institutions” in the sense of being non‐economic institutions that are considerably influenced by economic phenomena, and “economically relevant institutions” in the sense of being non‐economic institutions that influence economic phenomena (see also Swedberg, 2005).

Consecrating the business and corporate elites as the Swedish monarch does in his role as head of state, essentially appearing as an economic institution that acts to secure its own interests, as in the case of the British and Thai monarchies as well (see Clancy, 2021; Unchanam, 2020), or trying to constantly be useful from an economic point‐of‐view (see Billig, 1992), are not only of value to neoliberalism and its apostles; these activities can also be seen as expressions through which monarchies try to reinvent themselves in order to remain legitimate, which has been a constant quest throughout history (cf., Cannadine, 2012; Hobsbwam, 2012). Indeed, today we live in the neoliberal, market‐oriented era, thus monarchies try to embrace and accommodate a neoliberal appearance and behavior, with more or less enthusiasm and intensity. Obviously, even though a modern monarch's legitimacy still ultimately relies on blood and inheritance, this is not enough for their reproduction as the foremost representatives of an elite class (cf., Friedman & Reeves, 2020). To this extent, consecrating business and corporate elites may have less to do with any particular expression of political ideology on the part of a monarch, but more to do with pragmatism and survival.

Some might argue that the observations proposed in this paper are relevant for a Swedish or perhaps Scandinavian context only, implying that these countries are peculiar through their long social‐democratic traditions that have emphasized social and income equality and welfare for all. While this is true to some extent, such monarchies as the United Kingdom, Canada and Australia are today not very different structurally from Sweden or the two other Scandinavian monarchies, Denmark and Norway. Indeed, through intensive deregulations and privatizations and other expressions of neoliberalism, Scandinavia has become more similar to the Anglo‐Saxon world during the last decades (see Aaberge et al., 2018; Piketty, 2020). As a result, the observations in this paper should offer some relevant general insights related to the way business and their elites have come to dominate contemporary society, including monarchies, as part of the global triumph of neoliberalism. The way monarchies legitimate their privileges and unique access to resources by consecrating business, rather than resorting to traditional ideas of class and inheritance, not only contributes to their reproduction, but to their control and manipulation of their environments as well. Hence, royals' consecration has a dual function: to integrate business and corporate elites as part of a closely‐knit and opaque power elite, and to exercise power according to their own social, political and economic interests and perspectives.

## Data Availability

The data that support the findings of this study are available from the corresponding author upon reasonable request.

## ENDNOTES

^1^
For instance, for the official category “friends” among invitees to the King's 70th anniversary, I retrieved information about them through public Swedish databases, such as Proff, Allabolag, and Ratsit.

^2^
The Swedish monarchy is funded by Swedish tax payers (ca. 15 M Euros yearly), which should cover both the expenses of the members of the royal family, including their “private” expenses (such as clothes, travels during vacations, etc.), and the maintenance of royal properties. All palaces and their furniture etc., are owned by the state, but are maintained by the royal court. In addition to the public funding, the royal court exerts a number of business activities that contribute to its funding, such as parking fees, leases of houses and apartments, and cafés and shops. The private fortune of the royal family is not disclosed and therefore not known.

^3^
This committee is chaired by a senior court official and consists of three other members: a senior representative of Swedish business, a senior representative of the Swedish culture sector and academia, and the Head of Protocol of the Ministry for Foreign Affairs. The latter participates as the King occasionally offers royal medals to foreign heads of states, for example, on a state visit. Hence, two sectors in Swedish society are represented in the awarding committee through two individuals from outside the Royal Court: business, and culture and research/higher education.

^4^
These are known in Swedish as *H M Konungens medalj av 12:e storleken i serafimerordens band* and *H M Konungens medalj av 12:e storleken i högblått band*. Excluded from my examination are the even more prestigious *Serafimermedaljen*, only given to four people during the period and the *H M Konungens medalj av 12:e storleken med kedja*, only given to 28 people during the studied years. All data on the medals come from the Royal Court, see particularly the “Hovkalendern 2020”; see also Holmqvist (2023).

^5^
I exclude 42 of those, which were given to senior members of the royal court as an appreciation for their long‐time services to the Royal Family,

^6^
The data comes from “Sveriges statskalender” for each year; see also Holmqvist (2023).

^7^
The King also hosts other regular dinners, such as “Gala Dinners” on the occasion of state visits from abroad, a dinner every fourth year for the members of Parliament and the annual “Nobel Dinner,” in honor of the Nobel Laureates. I have, however, chosen not to include them.

^8^
The years were chosen to offer not only a snapshot observation of the invitations to royals dinners from 1 or 2 years, but to offer more systematic observations from several years.

^9^
I have excluded high‐ranking politicians (the prime minister, ministers in the government, secretaries of state) and senior government officials such as director generals, senior judges and prosecutors, who are normally always invited to the National Dinners for formal reasons as they are representatives of the state. For the category “Business,” corporate managers, capital owners and bankers dominate; for the category “Academy,” university presidents dominate; for the category “Culture,” heads of national theaters, operas and museums dominate; and for “Religion,” bishops and similar religious elites dominate. The category “Media” consists of senior editors and journalists.

^10^
The years for the Sweden Dinners were, as was the case for the National Dinners, chosen to offer not only a snapshot observation of the invitations from 1 or 2 years, but to offer more systematic observations from several years.

^11^
The categories presented consist of local elites in business, culture and academia. The other categories (not shown in the figure) are local elites in sports (4.5%), municipality officials (3.5%), social welfare (2.5%), politics (2%), education (2%), media (0.5%), healthcare (0.5%), religion (0.5%). A category “Others” are diverse local elite groups, less than 0.5% each (in total 3%).

^12^
The category “Business” primarily consists of senior business leaders and executives.

^13^
The category “unclear” primarily consists of women with titles as “Countess,” “Duchess” and similar, and men with titles such as “Mister.”

^14^
The category “Politics” consists of party chairpersons, cabinet members, and the City of Stockholm officials.

^15^
In future studies of who gets invited to royal dinners, studying the hierarchy of table placement would be relevant, that is, in addition to analyzing which groups are invited, examining how different groups are placed in relation to the king and queen would offer further understanding of the importance of various elites in society (cf., Hjelbrekke & Korsnes, 2018). Furthermore, something that would also be interesting to examine in the future is which people are repeatedly invited to the King's various dinners, and what social spheres they represent.

^16^
The visits are the following: Germany (outgoing, 2016), India (outgoing, 2019), Ireland (outgoing, 2019), Botswana (outgoing, 2011), Brazil (outgoing, 2010), Iceland (incoming, 2018), Chile (incoming, 2016), Tunisia (incoming, 2015), South Korea (incoming, 2019), and Canada (incoming, 2017).

^17^
One should also take into consideration that the prime minister and other cabinet members regularly make official trips to promote Sweden's businesses and corporations as well.

^18^
This refers to incoming state visits from Romania, Luxembourg, Finland, Turkey, India, Chile, Iceland, and South Korea.
